# Frailty as a predictor of adverse outcomes in burn patients: a systematic review

**DOI:** 10.1186/s12877-023-04302-5

**Published:** 2023-10-19

**Authors:** Arman Shafiee, Razman Arabzadeh Bahri, Shahryar Rajai, Mohammad Ahoopai, Niloofar Seighali, Mohammad Javad Amini

**Affiliations:** 1 Clinical Research Development Unit, Alborz University of Medical Sciences, Karaj, Iran; 2 Student Research Committee, School of Medicine, Alborz University of Medical Sciences, Karaj, Iran; 3 School of Medicine, Tehran University of Medical Sciences, Tehran, Iran; 4 School of Medicine, Shahid Beheshti University of Medical Sciences, Tehran, Iran; 5 Student Research Committee, Tehran Medical Sciences, Islamic Azad University, Tehran, Iran

**Keywords:** Burn, Frailty

## Abstract

**Background:**

The identification of new prognostic tools for the prediction of burn patients’ morbidity outcomes is necessary. Considering the feasibility of frailty assessment in the clinical setting, we aim to systematically review the literature on the associations between frailty and adverse outcomes in burn patients.

**Methods:**

Studies were retrieved from MEDLINE (through PubMed), Web of Science, Scopus, and Embase from their inception up to 8 September 2022. Included studies were those that used frailty indices to predict adverse outcomes in burn patients. The quality assessment was done using the National, Heart, Lung, and Blood Institute (NHLBI) checklist. The results were synthesized narratively.

**Results:**

We included 18 studies. The sample size among the included studies varied between 42–1615 patients. There were 12 research articles and 6 conference abstracts. Most of the studies were recently published in 2021 and 2022. Seven different frailty measures were evaluated. The following frailty measures were used: Canadian Study for Health and Ageing (CSHA) Clinical Frailty score (CFS), Modified frailty index-11 (mFI-11), Hospital frailty index, FRAIL scale, Emergency General Surgery Frailty Index (EGSFI), and Burn frailty index (BFI). There was only one report regarding a specific frailty index designed for the burn population (BFI). Except for one study (which used mFI-11), all included studies have shown a significant effect between assessing frailty and predicting worse outcomes. The CFS was an independent predictor of mortality among the burn population with high certainty of evidence. We found a significant association for other frailty indices as a predictor of mortality, however, the certainty of evidence regarding those was not high. Eight studies found a positive association between assessing frailty and unfavorable discharge location. There was no association between frailty and increased length of stay.

**Conclusion:**

In conclusion, the postadmission assessment of frailty can be a reliable tool for predicting unfavorable outcomes and mortalities among patients with burn injuries. In addition, future studies with various populations from other countries are required to evaluate the efficacy of frailty indices measurement in order to strengthen the available evidence.

**Supplementary Information:**

The online version contains supplementary material available at 10.1186/s12877-023-04302-5.

## Introduction

Burn injuries usually lead to morbidities, reduced quality of life, and fiscal burdens on the patients and their families. Burns also raises the cost of healthcare services, as their treatment requires extensive follow-ups, accompanied by prolonged hospital stays and potential surgical interventions [1]. Prognostic risk factors of burn are stated by older age, wider total body surface area burn (TBSA), inhalational injury, mechanical ventilation, presence of tracheotomy, and time from of burn injury to BICU admission and initial centre of first emergency treatment [2]. Understanding the prognosis of a burn patient can help us determine their treatment protocols and patient care facilities. Numerous injury scoring systems are already at our disposal regarding burn patients, including but not limited to age, total body surface area burned, inhalation injury, size and depth of burn, and serum creatine kinase [3]. A study by Silva et al. [4] reported that elderly burn patients’ comorbidities have a prevalence of 53–68%. The patients are at greater risk of premature death as a consequence of injuries, including burn injuries [1, 5, 6]. In the study by Pham and colleagues [7] which was conducted using data from burn centers in the United States and Canada, the in-hospital mortality rate for 55–64, 65–74, and ≥ 75 years old of age was 8.7%, 16.2%, and 24.4%, respectively. The major limitation of age consideration is the fact that it does not predict the physiological or psychological status of the elderly population. Therefore, a practical tool must consider different aspects of an elder patient. In fact, frailty, which is a state of vulnerability of an individual, has increased due to age-related decline in the function of the body [8]. Previous studies from single centers with small sample sizes have demonstrated the effects of frailty on burn mortality and showed that frailty is associated with an increased risk of mortality [9–12].

Frailty, usually defined as conditions with excessive vulnerability in response to endogenous and exogenous stressors, has also been proposed as a valid tool for predicting adverse outcomes among burn patients, and there have been some articles recently published on this subject [9, 13–15]. There is no universal definition for frailty, although definitions have been described in some studies [16–19]. However, frailty is best defined as a balance between assets and deficits, where if the deficits outweigh the assets, the person is deemed frail [20]. These factors can be measured, and in turn, frailty itself can be measured by pooling the overall results of these measures. Frailty indices such as The Canadian Study of Health and Aging frailty index and The Modified Frailty index (MFI) are derived by this method. The Canadian Study of Health and Aging clinical frailty scale (CSHA-CFS) is a 7-point clinical opinion scale created in order to be used as a clinical alternative to The Canadian Study of Health and Aging frailty index [21]. The CFS is a validated measure for assessing one’s physical frailty [21]. This scale does not evaluate psychological or social domains. The scoring system starts with 1 (very fit) and ends with 9 (terminally ill). One can be frail if the overall score becomes 5 or higher. Like CFS, the mFI-11 only assess physical domains and there have been critiques on its low responsiveness to change [22, 23].

Systematic reviews have been conducted on prognostic factors regarding burn patients [3, 24]. However, no study has reviewed and examined the prognostic value of frailty in burn patients. Considering the feasibility of frailty assessment in the clinical setting, we aim to systematically review the literature on the associations between frailty and adverse outcomes in burn patients..

### Methodology

The present systematic review was conducted based on Preferred Reporting Items for Systematic Reviews and Meta-Analyses (PRISMA) guidelines and guideline retrieved from the Cochrane Handbook for Systematic Reviews of Interventions [25, 26]. The protocol of this review was prospectively registered on PROSPERO with the following registration code: CRD42022353197.

### Search strategy

We performed a comprehensive database search in international databases, including Medline (via PubMed), Embase, Scopus, and Web of science up to 8 September 2022. No limitation was implemented on our search results. Furthermore, by screening the reference section of the potentially included articles, eligible studies were identified. A combination of the following keywords and Boolean operators were used to design the strategy of our systematic search: Burns, Burning, Frailty, Frailness, Debility, and Frail. The detailed search strategy of each database with exact results and time of the performance is available in Supplementary material Table 1.

### Eligibility criteria

The inclusion criteria based on PICOT definition were: 1) Population: adult burn patients; 2) Index: evaluated frailty as a predictor of post admission outcomes using a well-established and validated frailty scale; 3) Comparison: not applicable; 4) Outcome: reported relevant outcomes including but not limited to mortality, length of stay, high level of care discharge, and etc.; 5) Type of Study: all types of original studies. The was no limitation on date and the language of the published report. Conference abstracts were also included but their quality was not assessed due to limited data available regarding their methodology. Review studies, case report studies, meta-analyses, commentary studies, and letter to editor articles without any relevant data were excluded.

### Screening and data extraction

Screening the articles were performed in 2 steps: 1) Initial screening by title/abstracts; and 2) Full texts screening. Disagreements were resolved through discussion. Data were extracted on an Excel spreadsheet. The extracted data included Author, Year, Country, Registry/ Duration, Population, Total patients, Frailty index used, Cut-offs, and Main Findings. A third reviewer checked both screening and data extraction parts.

### Quality assessment

The quality assessment was evaluated by two reviewers (S.R. and M.A.) using a checklist derived from National, Heart, Lung, and Blood Institute (NHLBI) tools for cohort and cross-sectional studies [27]. The questionnaire contains 14 signaling questions including Q1. Clarity; Q2. Clearly specified population; Q3. Participation rate > 50%; Q4. Similar population; Q5. Sample size justification, Q6. Exposure before outcome; Q7. Adequate timeframe; Q8. Different levels of exposure; Q9. Exposure measurement quality; Q10. Repeated exposure assessment; Q11. Outcome measurement quality; Q12. Blinded assessment; Q13. Lost to follow-up rate < 20%; and Q14. Through statistical analysis, including adjustment of confounding variables. Full description of each signaling question is available in Supplementary Material Table 2. The overall quality of a study was based on the overall judgment of authors who answered and evaluated each study. Any discrepancies in quality assessment were resolved by the third reviewer.

### Synthesis and certainty of evidence

Since there were a lot of heterogeneity in the ways of reporting the findings of each article, we decided not to perform a meta-analysis. To rate the evidence, we used the GRADE-pro website and its definitions for each domain. Further detail about the system of rating has been provided in our previous work and on the GRADE-pro website [28–30]. Briefly, to report the pooled results of the studies and evaluating the certainty of evidence available for each frailty index, we used the Grading of Recommendations Assessment, Development, and Evaluation (GRADE) system developed for systematic reviews by assessing different domains which includes: 1) Risk of bias: the overall results of the quality assessment of each study was used to determine if it is necessary to rate down the evidence. The results derived from conference abstracts was considered to have serious risk of bias; 2) Inconsistency: in cases where different results were available from different studies (for example one study reports favorable use of frailty index and the other study not), the evidence was decreased one level; 3) Indirectness: defined exactly as defined by the GRADE team; 4) Imprecision: having few number of studies reporting the relevant outcome would decrease the level of evidence; 5) Publication bias: defined exactly as defined by the GRADE team. The certainty of evidence starts from high and ends on very low. Issues in each domain will decrease the evidence one or two levels [31, 32].

## Results

### Characteristics

**Fig. 1:**
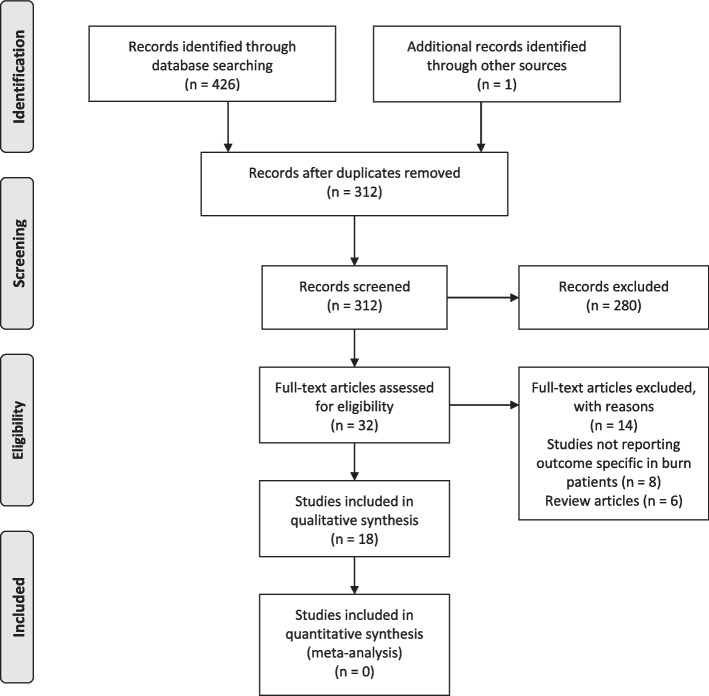
PRISMA flow diagram

Our search results yielded 426 articles, of which, 115 were duplicate. After screening based on title/abstracts and full texts, a total of 18 articles were included [9–15, 33–44] (Fig. 1). Data from one original article and abstract was the same [33, 34]. Included studies were published between 2013–2022. Twelve studies were journal articles and 6 were conference abstracts [33, 36, 39–42]. All studies were observational in terms of study design. Most of the studies were conducted in USA (*n* = 13), followed by UK (*n* = 4), and Turkey (*n* = 1). Data from one study was originated from The National Inpatient Sample (NIS) registry and 60,515 patients were included [43]. The participation rate of other studies varied between 42–1615 patients. All studies put an age limitation on their inclusion criteria, of which, most included 65 or older patients. The detailed characteristics of each included study with their main findings is available in Table 1.

**Table 1:** Characteristics of the included articles

ID	Author	Year	Country	Registry/ Duration	Population	Total patients	Frailty index	Cut-offs	Main Findings
1	Yi, Y. [43]	2022	USA	National Inpatient Sample/ 2016–2018	Patients ≥ 50 years of age with an acute burn diagnosis	60,515	Hospital frailty index	Low < 5; moderate 5–15; high > 15	1) Multivariable analysis for in-hospital mortality showed the OR [95% CI]: 3.62 [2.81–4.67] for the moderately frail group and 5.70 [3.60–9.02] for the highly frail group; 2) AUROCs for the model including Hospital frailty index: 0.84
2	Wolf Horrell, E. [40]	2021	USA	Single center/ 7 months	All admitted acute burn patients ages 45 and older	85	FRAIL Scale	Robust; pre-frail; frail	1) More palliative care consultations in the prefrail/frail group, although it was non-significant (*p* = 0.096); 2) Increased length of stay in the prefrail/frail group (*p* = 0.002); 3) Higher level of care discharge in the prefrail/frail group (*p* = 0.032)
3	Wearn, C. M. [33, 34]	2014	UK	Center based/ 9 years retrospective study	All burn admissions aged ≥ 65	228	Canadian Study for Health and Ageing (CSHA) Clinical Frailty score (CFS)	NA	1) CFS was significantly higher among non-survivors (*p* = 0. 010); 2) Multivariate analysis showed that the CFS is an independent predictor of mortality (*p* = 0. 043); 3) AUROCs analysis: 0.892; 4) Each increase in CFS point showed the OR [95% CI]: 1.588 [1.156–2.179] for mortality
4	Ward. [9]	2018	UK	Center based/ 8 years retrospective study	All patients > 65 years admitted to the burns center	239	Rockwood’s Clinical Frailty Scale	NA	1- Mortality: Frailty as an independent predictor of mortality *p* < 0.0001 2- In-hospital mortality: increased in-hospital mortality OR:2.33, *p* < 0.0001 3- One-year mortality: increased one-year mortality OR:3.13 (sensitivity = 83.9%) *p* < 0.0001 4- Spearman correlation coefficient (Rho) for in-hospital mortality: Rho = 0.31 (0.3–0.49 moderate association) *p* < 0.0001 5- Spearman correlation coefficient (Rho) for 1 year mortality: Rho = 0.5 (0.5 or more, large association) *p* < 0.0001
5	Wallace, D. L. [42]	2022	USA	Center based/ January 2016—December 2017	Burn patients	1615	Modified frailty index-11	NA	1- Not an independent predictor of mortality 2- Not an independent predictor of LOS
6	Wallace, D. L. [41]	2022	USA	Center based/ 2009–2018	Patients > 50 years with acute burn injuries	953	Canadian Study for Health and Ageing (CSHA) Clinical Frailty score (CFS)	NA	1- Predictor of mortality with OR = 2.9 (≥ 5 v 1–4) 2- Not associated with LOS *p* = 0.52 3- Predictor of discharge destination *p* < 0.0001
7	Sepehripour. [37]	2018	UK	Center based/ 2009–2010	Patients > 75 year sustaining a burn injury	129	Canadian Study for Health and Ageing (CSHA) Clinical Frailty score (CFS)	NA	1) Pearson correlation coefficient of *p* = 0.034 shows a strong correlation between frailty and complication (reduced mobility and cognitive impairment)
8	Romanowski. [38]	2020	USA	Center based/ 2008–2017	Burn patients ≥ 60 years old	83	Canadian Study for Health and Ageing (CSHA) Clinical Frailty score (CFS)	NA	1- Not a predictor of mortality (OR [95% CI] = 0.848 [0.492, 1.467], *p* = .554) 2- Not a predictor of LOS (estimate = − 0.496 [− 5.254, 4.262], *p* = .836)
9	Romanowski. [10]	2018	USA	Center based/ 2008–2013	Burn patients ≥ 50 years old	502	Canadian Study for Health and Ageing (CSHA) Clinical Frailty score (CFS)	NA	1- Independent predictor of mortality *p* < 0.001 2- Predictor of high level of care discharge destination *p* < 0.05 3- Multivariate analysis showed frailty is an independent predictor of mortality (OR [95% CI] = 1.94 [1.3 to 2.8]; [45]). Among patients aged 50–64, the estimate was OR [95% CI] = 2.5 [ 1.4–4.6] Among patients aged > 65, the estimate was lower (OR [95% CI] = 1.63[ 1.003–2.7])
10	Romanowski, K. S. [11]	2015	USA	Center based/ 2011–2013	Acute burn patients 65 years or older	89	Canadian Study for Health and Ageing (CSHA) Clinical Frailty score (CFS)	NA	1) Frailty scores were greater among non-survivors (5.2 ± 1.2 vs 4.4 ± 1.2) 2) Frailty scores were greater among those with poor discharged location (5.34 ± 0.9 vs 4.1 ± 1.2) 3) Multivariate analysis showed admission frailty scores are independently associate with poor discharge location 2.5 [1.3–4.8, 95% CI]) and mortality 1.67 [1.01–2.7, 95% CI])
11	Özlü, Ö. [15]	2022	Turkey	Center based/ 2017–2020	Hospitalized burn patients 65 years or older	67	Canadian Study for Health and Ageing (CSHA) Clinical Frailty score (CFS)	Normal: (CFS:1–3); Vulnerable: (CFS:4); Frail: (CFS:5–9)	1) CFS is an independent predictor of poor prognosis (those died or had major amputation) OR [95% CI] = 6.7 [0.8–55.6] for vulnerable group and OR [95% CI] = 47.8 [6.5–340.6] for frail group; *p* = 0.0001
12	Maxwell, D. W. [36]	2018	USA	Center based/ 2013–2017	Burn patients 65 years or older	100	Emergency General Surgery Frailty Index (EGSFI)	NA	Frail patients showed more 1) complications (61.8% vs 10.6%) (*p* < 0.05); 2) non-home discharges (67.6% vs 13.6%), 3) ICU admissions (52.9% vs 10.6%), 4) longer ICU stays (17 ± 23.0 vs 1 ± 7.0 days), and 5) in hospital mortalities (11.8% vs 1.5%)
13	Masud, D. [12]	2013	UK	Center based/ 2005–2009	Burn patients 65 years or older	42	Canadian Study for Health and Ageing (CSHA) Clinical Frailty score (CFS)	NA	1) Mortality: significantly lower frailty scores Median IQR 3 (2–7) compared to the non-survivors Median 5 (*p* = 0.0001) 2) Optimal cut off value for frailty score for mortality is > 3 (sensitivity = 100%, specificity = 72%) 3) Multivariate analysis showed for every 1-point increase in the frailty score the probability of death increases by 2.1 [95% CI] = 1.0798–4.0480)
14	Madni, T. D. [35]	2018	USA	Center based/ 2009–2014	Burn patients 65 years or older	126	Canadian Study for Health and Ageing (CSHA) Clinical Frailty score (CFS)	NA	1) The mean (SD) regarding the frailty score was significantly higher in those needed goals of care discussion (4.7 ± 1.3 vs. 3.8 ± 0.95; *p* = 0.0006), OR [95% CI] 3.42 ([1.54–7.60]; 2) The mean (SD) regarding the frailty score was significantly higher in those with unfavorable disposition (5.0 ± 1.3 vs. 33.6 ± 0.80; *p* = 0.0001), OR [95% CI] = 9.01 [ 3.91–20.78]
15	Iles, K. A. [14]	2022	USA	Center based/ 2015–2019	Burn patients 65 years or older	652	Canadian Study for Health and Ageing (CSHA) Clinical Frailty score (CFS)	Low (1–3), medium (4–6), or high (7–9)	1) Mortality: greatest in the high frailty group 24.3%, 7.0%, and 2.3%. Hazard ratios comparing high vs. low frailty group and high vs. moderate frailty group were 5.73; 95% CI (1.86, 17.62) and 2.19; 95% CI (0.87–5.50), respectively; 2) Median LOS was similar between subgroups (median 6–9 days); 3) ICU stay: greatest in the high frailty group (68% vs 37% and 21%, *p* < .001); 4) Need for mechanical ventilation: greatest in the high frailty group (27% vs 19% and 8%, *p* < .001), compared to moderate and low frailty
16	Galet, C. [13]	2022	USA	Center based/ 2009–2019	Burn patients 50 years or older	851	Canadian Study for Health and Ageing (CSHA) Clinical Frailty score (CFS)	Non frail < 5, frail > = 5	Multivariate analysis showed frailty was associated with increased: 1) Acute respiratory failure (OR [95% CI] = 2.599 [1.460–4.628], *p* = .001); 2) Mortality (OR [95% CI] = 6.080 [2.316–15.958]; *p* < .001); 3) Poor discharge disposition (OR [95% CI] = 3.135 [1.784–5.508], *p* < .001)
17	Andre, J. A. [39]	2021	USA	Secondary analysis from Transfusion Requirement in Burn Care Evaluation (TRIBE) study/ 2021	Burn Population	347	Modified frailty index-11/ Modified frailty index-5	Frail: MFI > 1 on either scale	1) As continuous variable, MFI-5 (OR [95% CI] = 1.86; [1.11–3.11]; *p* = 0.02) and MFI-11 (OR [95% CI] = 1.83[ 1.18–2.8]; *p* = 0.007) were independent predictors of mortality 2) MFI-11 > 1 was an independent predictor of mortality (OR [95% CI] = 2.91; [1.1–7.7]; *p* = 0.03); whereas, MFI-5 > 1 was not (OR [95% CI] = 2.6[ 0.95–7]; *p* = 0.06)
18	Maxwell, D	2019	USA	Center-based/ February 10, 2011 to June 8, 2017	Burn patients > 65-years	100	Burn frailty index	Frail > = 0.30	1- Mortality: 12 occurred in frail group, 1 occurred in non-frail 2- Median length of stay for not frail and BFI frail patients was 5 days (range 1–67) and 15 days (range 1–96), respectively 3- Sensitivity and specificity of the BFI predicting all-cause mortality is 0.923 (95% CI = 0.621–0.996) and 0.771 (95% CI = 0.608–0.807) 4- Patients classified as frail had significantly more complications (*p* < 0.001), non-home discharges (*p* < 0.001), ICU admissions, and longer hospital and ICU lengths of stay (*p* < 0.001), decreased 1 and 3-year survival (*p* = 0.001)

### Summary of frailty indices used

Among the included studies, there were 7 different frailty measures used for assessing the prognostic value of them among burn population. The most used frailty measure was the one developed by the Canadian Study for Health and Ageing (CSHA) Clinical Frailty score (CFS) (64%) [9–15, 33–35, 37, 38, 41]. Among the included studies, only 2 reported their outcomes among different frail groups [14, 15]. The second most used (11%) frailty index was the Modified frailty index-11 (mFI-11) [46]. Both studies which have used mFI-11 were conference abstracts [39, 42]. Each of the mentioned measures was used once within the reviewed studies: the Modified frailty index-5 (mFI-5) [39]. Hospital frailty index [43], FRAIL scale [40], Emergency General Surgery Frailty Index (EGSFI) [36], and Burn frailty index (BFI) [44]. The burn frailty index was developed based on the previous validated tool EGSFI.

### Frailty and postadmission outcomes

Except for one study [42], all included studies have shown a significant effect between assessing frailty and predicting worse outcomes following the admission of burn patients. Fifteen of the included studies, assessed the use of frailty as an independent predictor of mortality [9–15, 33, 36, 38, 39, 41–44]. Only two studies by Wallace [42] and Romanowski [38] reported a non-significant association. Based on our synthesis, the CFS was an independent predictor of mortality among burn population with high certainty of evidence (Table 3). Each of the following measures was assessed once with regard to predicting the odds of mortality: positive association of Hospital frailty index, FRAIL scale, EGSFI, and BFI. Regarding mFI-11 and mFI-5, there were heterogeneous results regarding their positive association [39, 42]. Considering other outcomes, unfavorable discharge location and length of stay were among the most reported, respectively. All eight studies found a positive association between assessing frailty and unfavorable discharge location [10, 11, 13, 35, 36, 40, 41, 44]. Regarding length of stay, only BFI and FRAIL scale showed significant increase in length of stay among prefrail/frail group by assessing frailty using FRAIL scale [40, 44]. Maxwell et al. found a decrease in length of stay among those whose were assessed as frail by BFI index [44]. All other studies did not find any association regarding frailty and increased length of stay. The detailed results of other outcomes are available in Tables 1 and 2.

**Table 2:** Summary results of synthesis and certainty of evidence

Outcome	No. of Studies based on each scale	Effect Estimate (In favor/ not in favor)	Risk Of Bias	Inconsistency	Indirectness	Imprecision	Publication Bias	Certainty of Evidence^b^
Mortality	CFS: 10	9/1	Not serious	Not serious	Not serious	Not serious	Not serious	High
	Hospital frailty index: 1	1/0	Not serious	NA	Not serious	Very serious	Not serious	Low
	mFI-11: 2	1/1 (345 patients/ 1615 patients)	Serious	Serious	Not serious	Serious	Not serious	Very low
	mFI-5: 1	0/1	Serious	NA	Not serious	Serious	Not serious	Very low
	EGSFI: 1	1/0	Not serious	NA	Not serious	Very serious	Not serious	Low
	Burn frailty index: 1	1/0	Not serious	NA	Not serious	Very serious	Not serious	Low
Length of stay	CFS: 3	0/3	Not serious	Not serious	Not serious	Serious	Not serious	Moderate
	FRAIL Scale: 1	1/0	Serious	NA	Not serious	Very serious	Not serious	Very low
	mFI-11: 1	0/1	Serious	NA	Not serious	Very serious	Not serious	Very low
	Burn frailty index: 1	1/0	Not serious	NA	Not serious	Very serious	Not serious	Low
Poor discharge^a^	CFS: 5	5/0	Not serious	Not serious	Not serious	Serious	Not serious	Moderate
	FRAIL Scale: 1	1/0	Serious	NA	Not serious	Very serious	Not serious	Very low
	EGSFI: 1	1/0	Not serious	NA	Not serious	Very serious	Not serious	Low
	Burn frailty index: 1	1/0	Not serious	NA	Not serious	Very serious	Not serious	Low
ICU stay	CFS: 1	1/0	Not serious	NA	Not serious	Very serious	Not serious	Low
	EGSFI: 1	1/0	Not serious	NA	Not serious	Very serious	Not serious	Low
	Burn frailty index: 1	1/0	Not serious	NA	Not serious	Very serious	Not serious	Low
Need for mechanical ventilation	CFS: 1	1/0	Not serious	NA	Not serious	Very serious	Not serious	Low
Goals of care discussion	CFS: 1	1/0	Not serious	NA	Not serious	Very serious	Not serious	Low

^a^Poor or unfavorable discharge is the discharge to skilled nursing facility

^b^Not Serious: no downgrade in the certainty of evidence; Serious: one downgrade in the certainty of evidence; Very Serious: two downgrades in the certainty of evidence; NA: not applicable

### Quality of the included studies

Among the journal articles assessed regarding their quality, one rated Poor, five rated Fair, and six rated Good. The detailed results of our quality assessment are available in Table 3.

**Table 3:** Quality assessment

Study id	1	2	3	4	5	6	7	8	9	10	11	12	13	14	Total assessment
Yi-2022 [43]	Yes	Yes	Yes	Yes	No	Yes	Yes	Yes	Yes	No	Yes	No	Yes	Yes	Good
Ward-2018 [9]	Yes	Yes	Yes	Yes	No	Yes	Yes	No	Yes	No	Yes	NA	Yes	Yes	Good
Sepehripour-2018 [37]	Yes	Yes	No	Yes	No	Yes	Yes	No	Yes	No	Yes	No	Yes	No	Poor
Romanowski-2020 [38]	Yes	Yes	No	Yes	No	Yes	Yes	No	Yes	No	Yes	CD	Yes	No	Fair
Romanowski-2018 [10]	Yes	Yes	Yes	Yes	No	Yes	Yes	No	Yes	No	Yes	No	Yes	Yes	Good
Romanowski-2015 [11]	Yes	Yes	No	Yes	No	Yes	Yes	No	Yes	No	Yes	No	Yes	Yes	Fair
Ozlu-2022	Yes	Yes	No	Yes	No	Yes	Yes	Yes	Yes	No	Yes	No	Yes	Yes	Fair
Masud-2013 [12]	Yes	Yes	No	Yes	No	Yes	Yes	No	Yes	No	Yes	No	Yes	Yes	Fair
Madni-2018 [35]	Yes	Yes	No	Yes	Yes	Yes	Yes	Yes	Yes	No	Yes	No	Yes	Yes	Good
Iles-2022 [14]	Yes	Yes	Yes	Yes	No	Yes	Yes	Yes	Yes	No	Yes	No	Yes	Yes	Good
Galet-2022 [13]	Yes	Yes	Yes	Yes	No	Yes	Yes	Yes	Yes	No	Yes	No	Yes	Yes	Good
Maxwell-2019 [44]	Yes	Yes	No	Yes	Yes	Yes	Yes	No	Yes	No	Yes	No	Yes	Yes	Fair

Questions: Q1. Clarity; Q2. Clearly specified population; Q3. Participation rate > 50%; Q4. Similar population; Q5. Sample size justification, Q6. Exposure before outcome; Q7. Adequate timeframe; Q8. Different levels of exposure; Q9. Exposure measurement quality; Q10. Repeated exposure assessment; Q11. Outcome measurement quality; Q12. Blinded assessment; Q13. Lost to follow-up rate < 20%; and Q14. Through statistical analysis (including adjustment of confounding variables)

## Discussion

In the present systematic review, we evaluated different frailty indices among burn patients. Also, we conducted a comprehensive assessment in the published articles regarding this topic. Seven different frailty indices were used in the included studies, including CFS, mFI-11, mFI-5, hospital frailty index, EGSFI, FRAIL scale, and BFI. The mostly used frailty indices were the CFS and mFIs, respectively. However, data regarding the association between frailty measurement as a prognostic tool among burn patients is limited. Most of the included studies showed that there is a positive correlation between measurement of frailty and the prediction of severe and comorbid outcomes among burn patients during their hospitalization. Although there is only one developed index to assess frailty among burn patients (BFI), based on our assessments, results obtained from the CFS index had a higher certainty of evidence score compared with other frailty indices, suggesting that it can be used as a reasonably reliable index than other indices. Our results yielded that measuring CFS as among admitted burn patients was able to predict mortality and poor discharge location. Furthermore, there were no significant association between measuring CFS and the length of hospital stay. It is worth mentioning that BFI was designed based on a previous validated frailty scale (EGSFI). Most of the included studies assessed the frailty scales for their patients at the time of admission because of the importance to predict burn outcome and status as soon as possible. Based on our results showing prognostic property of frailty assessment, it seems to be important to use standardized frailty scales (such as CFS and mFIs as common scales) for all burn patients at time of admission to predict their mortality, unfavorable discharge location, and length of stay.

During the initial period of admission with the busy and complicated condition of initial treatment of burn patients, the Frailty Score is simple to assess, understand, and communicate with clear criteria based on determined levels of patient function. Assessment of the risk of mortality and any worse outcome allows burn care teams to evaluate the impact of the primary hospital care and follow-up on burn outcomes.

In a study by Maxwell et al. [44], the association between burn-related complications and frailty was assessed using burn frailty index (BFI). It is worth mentioning that BFI was designed based on a previous validated frailty scale (EGSFI) which was previously validated for the burn patients as a useful tool for predicting morbidity and mortality [36]. However, using BFI was associated with better sensitivity and specify for predicting all cause mortality (0.923 and 0.771 vs. 0.846 and 0.736). A major concern regarding the burn frailty index is the fact that it can only be completed by patients who are able to fill the questionnaire, due to some questions such as questions about personal feelings. The CFS has an advantage over the burn frailty index because it can also be assessed using medical records or from the family members. Rockwood et al. [21] described a cumulative deficit model which is a model encompassing social, comorbidities, and cognitive factors. These factors increase the frailty index when combined. It has been reported in a systematic literature review by Shamliyan and colleagues [47] that frailty is associated with poor life expectancy. However, Sepehripour et al. [37] showed that clinical frailty scale was not correlated with higher or lower life expectancy in elder burn trauma survivors. Moreover, they saw a positive correlation between complications and frailty status.

Previous studies have been conducted in order to assess the predictors of mortality and other severe outcomes among burn patients [48–50]. However, there is no study which comprehensively assess the available evidence regarding the association of frailty and outcomes following burn injury. Age, %TBSA, % full thickness burn, female gender, inhalation injury, surgery including escharotomy, and the depth of burn were among the factors that has been reported to predict the length of stay among these patients [48]. In our study, however, the evidence suggests there were no association between frailty as a predictor of length of stay. Only one study which used BFI was reported a significant increase in the length of stay among frail patients [44]. Regarding health-related quality of life, a systematic review by Spronk et al. reported several factors including but not limited to severity of injury, depression, and post-traumatic stress symptoms as an independent predictor [50]. There were no studies reporting the predictive value of frailty on qualityof life following burn injury. A recent systematic review and meta-analysis have evaluated the available risk models for predicating mortality among burn patients [49]. They found the classic Baux; the revised Baux; and the Fatality by Longevity, APACHE II score, Measured Extent of burn, and Sex (FLAMES) among the best predictors of mortality. It is worth noting there was no discussion regarding measurement of frailty as a potential predictor of mortality in their systematic review [49].

There are several strengths regarding the present study. We performed a comprehensive bibliometric database search in order to identify all published articles in any language evaluating frailty indices on burn injury patients. We have also included conference abstracts to maximize the validity of our results. Also, there is no other systematic review or a meta-analysis on elderly burn patients. We fully screened the included studies and reported their data as several individual outcomes. Furthermore, we assessed the certainty of evidence in both outcomes and frailty indices used. Our study has some limitations. On one hand, a meta-analysis could not be performed in this systematic review, mainly due to the heterogeneity of the studies and low available data. On the other hand, most of our data were regarding the CFS index and there is low certainty of evidence about other frailty indices. Finally, the subjectiveness of our methodology using the GRADE system to synthesis our results may cause bias to our findings. Therefore, we suggest performing additional studies specifically on the frailty masseurs which have low or very low certainty of evidence regarding their use in burn patients.

## Conclusion

In conclusion, based on the results of the present systematic review, assessment of frailty can be developed and be used as a predictive tool for mortalities among the patients with burn injuries. In addition, more studies with various populations from other countries are also required to evaluate the efficacy of frailty measurement. Although there are considerable evidence indicating CFS as a great tool regarding this goal, future studies are needed to assess other frailty indices which were explained in details in this systematic review.

### Supplementary Information

**Additional file 1: Supplementary Table 1.** PRISMA 2020 checklist.** Supplementary Table 2.** Search strategies for online databases.

## Data Availability

All data has been presented in the manuscript.
